# Myokine Regulation of Insulin Secretion: Impact of Inflammation and Type 2 Diabetes

**DOI:** 10.3389/fphys.2019.01608

**Published:** 2020-01-22

**Authors:** Alexander J. Ryan, Theodore P. Ciaraldi, Robert R. Henry

**Affiliations:** ^1^Veterans Affairs San Diego Healthcare System, San Diego, CA, United States; ^2^Division of Endocrinology and Metabolism, Department of Medicine, University of California, San Diego, LA Jolla, CA, United States

**Keywords:** inflammation, myokines, skeletal muscle, type 2 diabetes, insulin secretion

## Abstract

Skeletal muscle (SkM) secretes protein factors (myokines) that can exert multiple actions. To study the control of myokine regulation of β-cell function, SkM biopsies were taken from non-diabetic (ND) and Type 2 diabetic (T2D) subjects and satellite cells cultured to myotubes (MT). MT were also treated with lipopolysaccharide (infectious inflammation – II) or a combination of glucose (10 mM), insulin (120 pM), and palmitate (0.4 mM) (metabolic inflammation – MI) to model the inflammatory and metabolic conditions seen *in vivo* with T2D. Conditioned media (CM) was collected from MT after 24 h and used to treat INS-1 cells for 24 h. Cell viability, total insulin content, glucose-stimulated insulin secretion (GSIS) and maximal (IBMX-stimulated) IS (IS_max_) were monitored. Under baseline conditions, CM from ND and T2D MT had no effects on INS-1 cell viability, insulin content, GSIS, or IS_max_. After exposure to II, CM from ND-MT augmented GSIS in INS-1 cells by 100 ± 25% over control (*p* < 0.05); T2D-CM had no effect. After exposure to MI, T2D-CM suppressed GSIS by 35 ± 5% (*p* < 0.05); ND-CM was without effect. Under either of these conditions cell viability, total insulin content and IS_max_ were unaffected. Effects of CM on GSIS were lost after CM was boiled. Both augmentation of GSIS by ND-CM from II-treated MT, and suppression by T2D-CM from MI-treated MT, were inhibited by wortmannin, Ro 31-8220, and SB203580. In summary: (1) ND-MT are able to augment GSIS when stressed, (2) T2D-MT responding to a diabetic-like environment secrete myokines that suppress GSIS, (3) Unknown protein factors exert effects specifically on GSIS, possibly through PI-3K, PKC, and/or p38 MAPK. In T2D, both insulin resistance and a suppression of adaptive increased insulin secretion are intrinsic properties of SkM that can contribute to the full T2D phenotype.

## Introduction

As the major insulin target tissue and the predominant site of post-prandial glucose disposal, insulin resistance in skeletal muscle (SkM) is a defining feature of type 2 diabetes (T2D) (DeFronzo and Tripathy, 2009). Beyond its role in regulated glucose disposal, evidence accumulated over the past 10–15 years has revealed that SkM can also be viewed as a secretory organ, releasing multiple factors (reviewed in De Rossi et al., 2000; Whitham and Febbraio, 2016). Proteomic analysis of the SkM secretome has identified several hundred potential protein myokines, as well as metabolites (Rai and Demontis, 2016), miRNAs (Massart et al., 2016), and other factors, often delivered as cargo in exosomes (Jalabert et al., 2016). While some of these secreted factors are unique to SkM (e.g., myostatin), many are cyto- and chemokines and growth factors produced and released from multiple tissues (reviewed in Raschke and Eckel, 2013). These muscle-secreted factors have been demonstrated to exert autocrine, paracrine and endocrine actions on multiple tissues (reviewed in Pedersen and Febbraio, 2012).

One such endocrine action of myokines and other muscle secreted factors could be to influence islet health and function, as shown by the impact of exercise. Exercise training programs improved both insulin sensitivity and glucose responsiveness of insulin secretion in sedentary individuals, with the nature of the response depending on the intensity of effort (Rohling et al., 2016). Given the fact that the expression and secretion of multiple factors, including myokines are regulated by exercise (reviewed in Pedersen and Febbraio, 2012), it is reasonable to expect that some of these factors may be involved in the β-cell response to exercise, though the specific factors and pathways involved remain to be investigated. One mechanism by which exercise can improve β-cell function was revealed by the work of Ellingsgaard et al. (2011), which showed that IL-6, either administered directly or increased following exercise, acted on intestinal L cells and pancreatic α-cells to stimulate GLP-1 secretion, ultimately elevating insulin secretion. A similar β-cell protective role of IL-6 was seen in exercising mice (Paula et al., 2015). Conversely, many myokines are pro-inflammatory and could contribute to islet inflammation. Evidence for such direct communication between muscle and the β-cell was provided by Bouzakri et al. (2011), where insulin resistance was induced in human myotubes (MTs) by treatment with TNFα. Exposure of isolated β-cells to conditioned media (CM) from these cells both increased apoptosis and reduced glucose-stimulated insulin secretion (GSIS) (Bouzakri et al., 2011), creating a model of β-cell failure. Only the later response was dependent on TNFα, suggesting that other muscle-secreted factors were responsible for modulating β-cell mass.

Seeing that the secretion of multiple myokines, e.g., TNFα (Saghizadeh et al., 1996), MCP1 (DiGregorio et al., 2005), and IL6 (Munoz-Canoves et al., 2013), as well as miR-29 (Massart et al., 2017) by T2D muscle or myotubes have been reported to be altered compared to healthy individuals or cells derived from them (Ciaraldi et al., 2016) (reviewed in Garneau and Aguer, 2019), it is possible that some of these factors could contribute to regulation of β-cell mass and function in T2D. The importance of insulin resistance in adipose tissue and SkM as initial “hits” in the pathogenesis of T2D cannot be overstated. However, since preserving β-cell function is also crucial to preventing diabetes (DeFronzo and Abdul-Ghani, 2011), it is critically important to understand how insulin resistant fat and muscle can contribute to β-cell failure and, conversely, how “healthy” fat and muscle may help preserve normal β-cell mass and function. With that background in mind, the current study was designed to address two specific questions: (1) How might the demonstrated differences in the secretome of SkM from T2D individuals impact β-cell function and mass, and (2) How might the environment that SkM is exposed to in T2D influence that secretome and it’s effects on the β-cell? We investigated these questions employing myotubes cultured from healthy and T2D individuals, which display differences in secretion of myokines and other factors, while also evaluating the impact of conditions designed to partially model the hormonal/metabolic milieu characteristic of T2D and the chronic, low-grade inflammation also observed in T2D (Olefsky and Glass, 2010).

## Materials and Methods

Cell culture materials were purchased from Irvine Scientific (Irvine, CA, United States) except for SkM growth medium and supplements, which were obtained from Lonza (Walkersville, MD, United States).

All other chemicals were reagent grade and purchased from Sigma Chemical (St. Louis, MO, United States). Electrophoresis reagents were from Bio-Rad (Richmond, CA, United States) or Invitrogen (Carlsbad, CA, United States). Primary antibodies were obtained from the following sources: IkBα (catalog #9242), phosphop38 MAPK (#9216), p38 MAPK (#8690), phospho-p44/42 MAPK (#4370), p44/42 (#4695), caspase 3 (#9665), phospho-S^473^-Akt (#4051), Akt (#4685) (Cell Signaling Technology, Beverly, MA, United States); phospho-JNK (#sc-6254), JNK (#sc-571) (Santa Cruz Biotechnology, Santa Cruz, CA, United States); β-actin (#NB600-503) (Novusbio, Littleton, CO, United States). Fluorescently labeled secondary antibodies and blocking buffer were obtained from Licor (Licor, Lincoln, NE, United States). Protein controls for caspase 3 (Jurkat cell extracts treated ± cytochrome C) were from Cell Signaling.

### Subjects

Samples of SkM were collected from 20 non-diabetic (ND) subjects and 22 T2D subjects. General inclusion criteria included: weight stable (± 2 kg) for 1 month and medication use stable for at least 3 months. Use of steroids and anti-depressants were cause for exclusion. Subjects were classified as ND based on fasting [glucose] < 100 mg/dL on screening and HbA1c < 5.7% within 2 months of biopsy. None of the subjects from the ND group had a family history of T2D. None of the women were taking hormonal replacement therapy. Blood was collected after an overnight (10–12 h) fast, serum prepared and stored at -80°C before analysis. Percutaneous needle biopsies of vastus lateralis muscle were performed and muscle tissue was immediately processed for culture.

### Cell Culture and Treatments

#### Skeletal Muscle Cells

The procedure for the isolation, propagation and differentiation of SkM cells has been detailed elsewhere (Ciaraldi et al., 1995). Briefly, after enzymatic isolation, muscle satellite cells were grown in serum-free SkGM (Lonza) supplemented with the bullet kit, omitting insulin. Cells were passed once into the required formats. After attaining 80–90% confluence, cells were fused for 5 days in α-MEM containing 2% fetal bovine serum (FBS), 100 U/mL penicillin and 100 mg/mL streptomycin. After three washes with PBS, the media was replaced with serum-free α-MEM (0.1% BSA) containing glutamine, antibiotics and the indicated treatments. The infectious inflammation (II) condition was induced by treatment with lipopolysaccharide (LPS, Sigma #4391), added from a 1 mg/mL stock in serum-free α-MEM to attain a final concentration of 1 μg/mL. The metainflammation (MI) mix was made fresh from stocks of D-glucose ([final] = 10 mM), human recombinant insulin ([final] = 120 pM) and palmitate conjugated to fatty acid-free BSA (Sinha et al., 2004) ([final] = 400 μM), diluted in serum-free α-MEM. CM was collected after 24 h in culture, centrifuged (800 × *g*, 10 min, 4°C) to remove debris and stored at −80°C. Fresh media and treatments were added to the muscle cells and these were cultured an additional 24 h before protein extraction (see below).

#### INS-1 Cell Culture

INS-1 cells were grown in RPMI 1640, supplemented with 10% FBS, 10 mM HEPES, 2 mM L-Glutamine, 1 mM sodium pyruvate, 50 μM β-mercaptoethanol, 100 U/mL penicillin and 100 mg/mL streptomycin. Cells were split when they reached 100% confluence, every 3–4 days. For experiments, INS-1 cells were seeded in 24-well plates, and were grown to 100% confluence. MT-CM was concentrated 2-fold using a Centricon filter (mw cut-off 3000), and a 3:1 mixture of RPMI:MT-CM (glucose supplemented to a final concentration of 11 mM) was used to treat INS-1 cells for 24 h before further experimentation. Individual wells were exposed to CM collected from a single subject, performed in duplicate.

#### Media Controls

**FIGURE 1:**
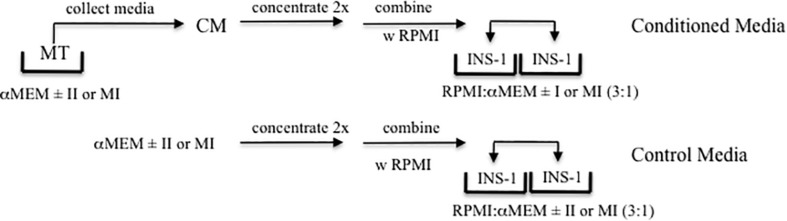
Experimental Design. Schematic representation of the production of MT conditioned (CM, top) and control (lower) media, and treatment of INS-1 cells.

Given that MT-CM was generated with a different medium (serum-free αMEM) than that in which INS-1 cells are routinely cultured (RPMI 1640-10%FCS), it was necessary to determine the conditions under which INS-1 cells could be exposed to MT-CM. Twenty four hour exposure of INS-1 cells to a 3:1 mixture of RPMI 1640 and s/f-α-MEM concentrated two-fold (glucose supplemented to attain a final concentration of 11 mM), resulted in no change in overall cell viability, as monitored by total cell protein, LDH release, or caspase 3 cleavage, when compared to RPMI 1640 alone (Supplementary Figure S1). More importantly, while there was a modest increase in total insulin content, there were no changes in insulin release in the presence of either low or high [glucose] (not shown). Consequently, there were no differences in GSIS or maximally stimulated IS (IS_max_) (Supplementary Figure S1). Moving forward, treatment of INS-1 cells with MT-CM refers to exposure to a 3:1 mix of RPMI1640 and two-fold concentrated s/f-α-MEM conditioned by MT. The control for this situation would be RPMI: αMEM (3:1) not conditioned by MT. For Figures 7, 8, the controls would be either RPMI: αMEM + II (3:1) not conditioned by MT or RPMI: αMEM + MI (3:1) not conditioned by MT. The nature of the controls specific to each set of experiments is also described in the figure legends. The protocol for the generation of CM and treatment of INS-1 cells is presented in Figure 1.

#### LDH Release Assay

Media was collected from MT and INS-1 cells after exposure to control or CM, centrifuged and stored at −80°. LDH release into the media was quantified using the “*In vitro* toxicology assay kit” (Sigma) following the manufacturer’s instructions.

#### Insulin Secretion

INS-1 cells were washed in HEPES-buffered salt solution (HBSS) and incubated for 1 h in HBSS containing 2.5 mM glucose. Cells were then stimulated with 2.5 mM, 16.5 mM glucose, or 16.5 mM glucose + 100 μM IBMX (for IS_max_). Each condition, high or low [glucose], was performed in duplicate, for each specific treatment. After 1 h the HBSS was collected, and the cells were lysed in extraction buffer (Ciaraldi et al., 2007). Insulin concentration in the media was calculated as a percentage of the total insulin content in that well. Insulin secretion index = [insulin]_high glucose_/[insulin]_low glucose._

#### Protein Extraction

After the collection of CM, muscle cells were rapidly washed 5× with 4°C PBS and then lysed in extraction buffer (Ciaraldi et al., 2007). Proteins were extracted from INS-1 cells as described in the insulin secretion protocol above. Protein concentration was determined by the Bradford assay and extracts stored at −80°C until analyzed.

#### Western Blotting

Cell protein extracts were resolved on 10% SDS-PAGE, transferred to nitrocellulose membranes and blocked overnight at 4° with Odyssey block (LI-COR Biosciences). Incubation with anti-bodies was for 3 h at RT. Detection and quantification of band intensity was performed using Odyssey Infrared Imaging System and Image Studio analysis software (version 3.1.4).

#### Assay of Circulating and Secreted Proteins

Serum insulin levels of the human subjects were determined with a human specific RIA kit (Millipore Corp, Billerica, MA, United States); sensitivity was 2 μU/mL, with inter- and intra-assay coefficients of variation (CV) of 6 and 4%, respectively, cross-reactivity with rat insulin = 0.1%. Secreted and cellular insulin from the rat INS-1 cells were detected with a rat specific RIA (Millipore); sensitivity of 0.1 ng/mL, CVs of 9 and 4%, cross-reactivity with human insulin = 100%. Selected myokines in serum and CM were analyzed with MILLIPLEX MAP kits (Millipore) using a BioPlex 200 instrument (Bio-Rad Corp, Hercules, CA, United States). Sensitivities (in pg/mL), inter- and intra-assay CVs for each analyte are as follows: IL1β (0.4, 7%, 6%), IL6 (0.3, 12, 8), IL8 (0.2, 12, 7), IL10 (0.3, 9, 5), IL15 (0.4, 10, 7), GRO (10.1, 12, 5), and VEGF (5.8, 8, 6). BDNF (sensitivity = 20 pg/mL, intra-assay CV = 4%) and TGFβ1 (sensitivity = 15.4 pg/mL, intra-assay CV = 3%) were measured using ELISA kits purchased from R&D Systems (Minneapolis, MN, United States). Wnt 3a was measured with an ELISA kit purchased from Lifespan Biosciences (Seattle, WA, United States) (sensitivity = 1.56 ng/mL, intra-assay CV = 5.4%), while Wnt 4 was measured by an ELISA kit from Ray Biotech (Norcross, GA) (sensitivity = 1.22 ng/mL, intra-assay CV < 10%).

### Statistical Analysis

Statistical analysis was performed using GraphPad Prism 8.0 (GraphPad, San Diego, CA, United States). Data were analyzed by *t*-test or 1 way ANOVA with Tukey’s *post hoc* test where appropriate. Data were tested for normality with the Kolmogorov–Smirnov test. For results that were not normally distributed, data were log-transformed for statistical analysis and then back-transformed and reported in original units as mean ± SEM. Statistical significance was accepted as *p* < 0.05. The number of individual determinations for each measurement is indicated in the Figure body or legend. An individual determination is a measurement made on MT or MT-CM from one individual subject, performed in duplicate (as indicated in the Figure legends).

## Results

### Subjects

Subjects were recruited from the general population by advertisement; their characteristics are presented in Table 1. All of the women but one were post-menopausal, that subject was biopsied during the early follicular phase of her cycle. None of the women were taking hormone replacement therapy. Assignment to the T2D group was made on the basis of an existing clinical diagnosis with the limitation of [HbA1c] = 7.5–9.5%. The time since diagnosis ranged from 1–15 years. T2D subjects continued on their prescribed medications up to the day of biopsy; sample collection was performed before morning medication. Anti-diabetic medication use included: insulin alone (*n* = 1), metformin alone (7), metformin + insulin (6), glipizide + metformin (1), glipizide + metformin + linagliptin (1), glipizide + metformin + insulin (1), canagliflozin + metformin (1), glipizide + metformin + insulin + linaglutide (1), liraglutide + metformin (1), liraglutide + metformin + insulin (1). One subject was controlled without medication.

**TABLE 1:** Subject characteristics.

	**ND**	**T2D**
n (F/M)	20 (2/18)	22 (7/15)
age (year)	57.0 ± 2.7	57.2 ± 1.7
BMI (kg/m^2^)	30.20 ± 1.04	33.27 ± 1.28
Fasting [Glucose] (mM)	5.30 ± 0.16	9.41 ± 0.81†
Fasting [Insulin] (pM)	55 ± 8	114 ± 19†
HOMA2-IR	1.61 ± 0.20	2.93 ± 0.55†
HOMA-%B	92.5 ± 8.7	63.3 ± 7.2*

*p < 0.05 vs. ND; †*p* < 0.01 vs. ND. Ave ± SEM.

The groups were similar in age, and BMI. The T2D subjects were significantly more insulin resistant and displayed reduced steady-state β-cell function (Table 1).

### Myokine Secretion

**FIGURE 2:**
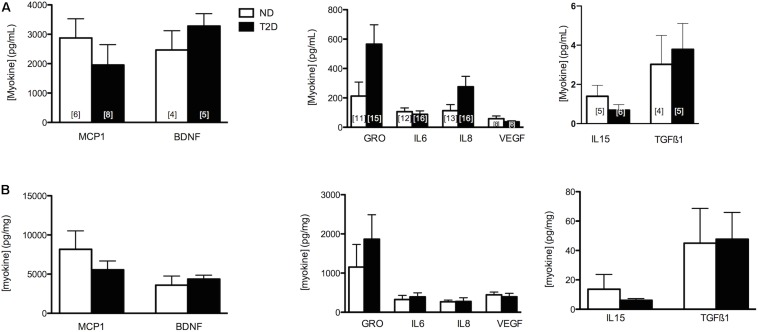
Myokine Secretion. Secretion of selected MK from ND- and T2D-MT. CM collected from untreated MT after 24 h in culture. Results presented as absolute value **(A)** or normalized to cell protein **(B)**, Ave + SEM. The numbers of sets of MT from individual subjects evaluated is presented within each bar, measured in duplicate.

The release of selected myokines from untreated MT, including several reported to influence β-cell mass or function (Nunemaker et al., 2014; Kozinski et al., 2016)(reviewed in Barlow and Solomon, 2018), was measured (Figure 2). While we reported previously that the secretion of GRO and IL8 was elevated from T2D-MT (Ciaraldi et al., 2016), and they both also tended to be higher with the current cohort, these differences did not attain statistical significance (*p* = 0.079 and 0.105, respectively), Neither Wnt3a nor Wnt4 could be detected in CM.

### Impact of ND and T2D Secretomes on INS-1 Cells and Insulin Secretion

**FIGURE 3:**
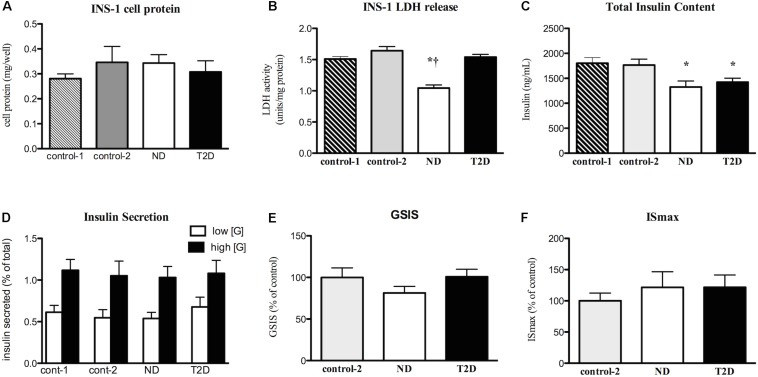
Effects of myotube (MT) conditioned media (CM) on INS-1 cell viability and function. Serum-free CM collected after 24 h and processed as described in the section “Materials and Methods.” INS-1 cells treated with the indicated media for 24 h before harvest or assay. **(A)** Total cell protein (*n* = 12). **(B)** LDH release (*n* = 12). **(C)** Total insulin content (*n* = 20). **(D)** Insulin secretion in the presence of low (2.5 mM) and high (16.5 mM) glucose (*n* = 11/11/16/12). **(E)** GSIS (*n* = 20). **(F)** Maximal, IMBX-stimulated, insulin secretion (IS_max_, *n* = 8). Results presented as absolute value **(A–D)** or as a percentage of Control-1 media **(E,F)**, Ave + SEM. Control 1 = RPMI, Control 2 = RPMI: αMEM (3:1) not conditioned by MT. “*n*” represents the number of independent determinations, CM from MT from separate individuals, each performed in duplicate. *p < 0.05 vs. controls, **†***p* < 0.05 vs. T2D.

Myotubes-Conditioned media would be expected to contain a stew of proteins, metabolites and other factors. CM collected from ND and T2D MT, when processed as described in section “Materials and Methods” had no negative impact on the measures of INS-1 cell viability over 24 h (Figures 3A,B and Supplementary Figure S1C) when compared to either RPMI 1640 (control 1) or the 3:1 mixture of RPMI+non-conditioned s/f-α-MEM (control 2). While total insulin content was reduced in cells exposed to either ND- or T2D-CM, compared to non-conditioned media, neither ND-CM nor T2D-CM altered other aspects of INS-1 cell function, including insulin secretion in the presence of either low or high [glucose] (Figure 3D). Consequently, GSIS and IS_max_ were comparable to what was seen in the presence of non-conditioned media (Figures 3E,F). There were no differences between the effects of CM collected from either ND or T2D-MT on any of these outcomes, including insulin content, but for LDH release, which was actually reduced by ND-MT-CM (Figure 3B).

### Impact of Infectious and Metabolic Inflammation on Myotubes

The control condition under which MT were differentiated reflects the euglycemia (5 mM) and normo-insulinemia (∼ 20 pM with 2% FCS) characteristic of healthy individuals. In studying the impact of inflammation on metabolic regulation, treatment with LPS has been used *in vitro* (Frisard et al., 2010; Kewalramani et al., 2010) to induce an inflammatory state. The doses of LPS most frequently used (0.1–1.0 μg/mL) are more reflective of those present during infection (infectious inflammation – II); we employed a similar dose. We also wished to determine the effects of a metabolic environment at least partially resembling that chronically present in the circulation of individuals with T2D. To establish these conditions, we determined the circulating fasting glucose, insulin and FFA levels of 24 consecutive T2D volunteers studied on our unit. None of these subjects were included in the present study. The average values were used to define the milieu used to induce metabolic inflammation (MI): [glucose] = 10 mM, [insulin] = 120 pM, [FFA] = 550 μM. Since palmitate represents the most abundant circulating FFA, we settled on 400 μM as a properly representative concentration, a level that has also been shown to induce insulin resistance *in vitro* (Hommelberg et al., 2011). That the fasting glucose and insulin concentrations of the current cohort of T2D subjects (Table 1) are similar to those in the MI mix validates this approach.

Exposure of either ND or T2D-MT for 24 or 48 h to either II or MI under serum-free conditions had no significant effect on total cell protein or cleavage of caspase 3 compared to s/f α-MEM lacking the additions (Supplementary Figure S2).

### Possible Inflammation in MT

**FIGURE 4:**
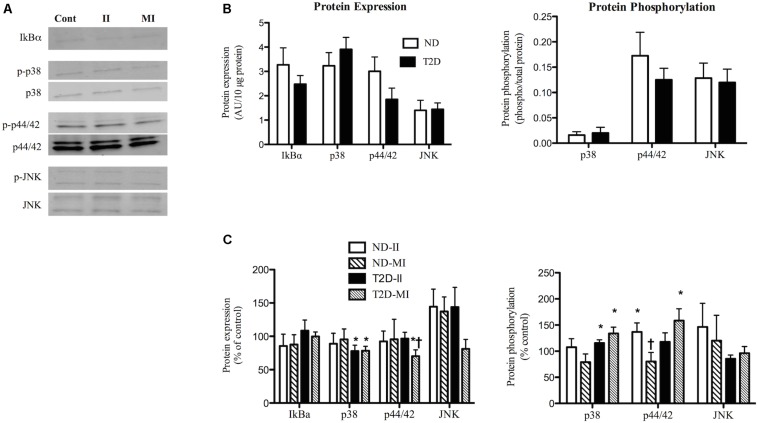
Effects of II and MI conditions on markers of inflammatory signaling in ND- and T2D-MT. **(A)** Representative western blots for IkBα, total and phosphorylated p38, p44/42, and JNK after MT treated for 48 h. **(B)** Quantification of western blots under control conditions (*n* = 10–13). **(C)** Quantification of western blots after treatment (*n* = 10–13). Results presented as absolute value **(B)** or as a percentage of the appropriate control media **(C)**, Ave + SEM. Control = MT from the same individual w/o treatment. *p < 0.05 vs. matched control, †*p* < 0.05 vs. matched II.

When cultured in s/f-αMEM with no additions there were no differences between ND and T2D-MT with regard to protein expression of IkBα, a marker of inflammatory state (Figure 4B). Similarly, there were no significant differences between the groups for either the protein content or phosphorylation of a number of key kinases involved in inflammatory signaling, p38-MAPK, p44/42-MAPK, and JNK (Figure 4B).

Exposure of ND-MT to the II condition for 48 h had no effect on the protein expression of IkBα, p38, p44/42, or JNK (Figures 4A,C). Phosphorylation of p44/42 in ND-MT was modestly increased by II (Figure 4C), while that of p38 and JNK were unaltered. T2D-MT displayed a different response to the II condition; p38 protein was down-regulated (Figure 4C), while its phosphorylation was modestly, but significantly (*p* = 0.044), increased.

Exposure of ND-MT to MI conditions had no significant effects on the protein expression or phosphorylation of the markers of inflammation (Figure 4C). T2D-MT showed greater sensitivity to MI, as protein expression of both p38 and p44/42 were reduced slightly, but significantly (Figure 4C), even as phosphorylation of both kinases was increased modestly.

There were several instances where responses of the cells from the same individual to II and MI conditions differed. In ND-MT, p44/42 phosphorylation increased after II and decreased after MI exposure (*p* < 0.01).

### Insulin Signaling in Myotubes

**FIGURE 5:**
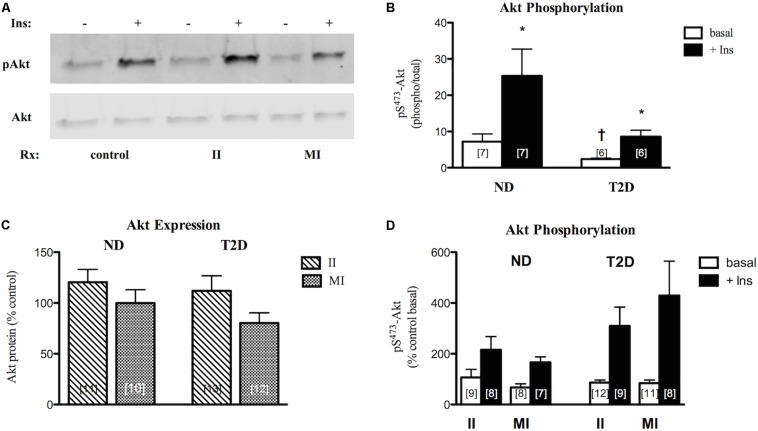
Effects of II and MI conditions on MT function. **(A)** Representative western blots for total and phosphorylated (S^473^) Akt after MT treated for 48 h. **(B)** Quantification of western blots for pS-Akt under control (w/o treatment) conditions. **(C)** Regulation of Akt protein expression by treatment. Results presented as a percentage of MT from the same individual w/o treatment. **(D)** Regulation of Akt phosphorylation. Results presented as a percentage of basal activity (phospho/total) in MT from the same individual w/o treatment, Ave + SEM. *p < 0.05 vs. basal, †*p* < 0.05 vs. ND.

Under control (no additions) conditions Akt protein expression was similar in ND- and T2D-MT (6.32 ± 0.65 vs. 7.97 ± 1.47 AU). In agreement with previous reports (Cozzone et al., 2008; Kase et al., 2015), both basal and insulin-stimulated phosphorylation of Akt on S^473^ were lower in T2D-MT (Figure 5B), though only the difference for basal activity attained statistical significance.

In ND-MT, neither II nor MI conditions had a statistically significant effect on Akt protein expression (Figure 5C). Again, T2D-MT seemed more sensitive to the MI condition, as the response of Akt protein expression to the two treatments did differ significantly (*p* = 0.019 for II vs. paired MI).

Both basal and insulin-stimulated Akt phosphorylation were unaltered in ND-MT treated under II conditions; insulin action was retained (*p* = 0.024 basal vs. insulin). Meanwhile, insulin stimulation was reduced, and no longer significant, after MI treatment (*p* = 0.056 basal vs. insulin) (Figure 5D), indicating an induction of insulin resistance. Neither basal nor insulin-stimulated Akt phosphorylation in T2D-MT were significantly influenced by either II or MI treatment; significant insulin responsiveness was retained (*p* = 0.016 and 0.023, basal vs. insulin, respectively).

### Regulation of Myokine Secretion by Infectious and Metabolic Inflammation

**FIGURE 6:**
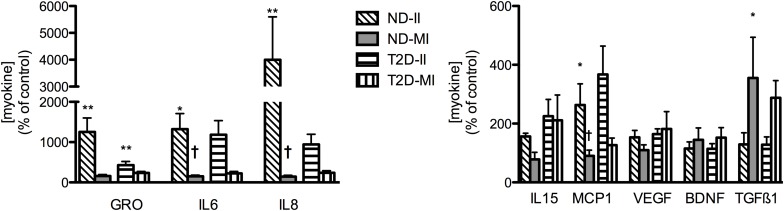
Regulation of myokine secretion by treatment with II or MI. Results presented as a percentage of the matched control (no addition) CM from MT from the same individual, Ave + SEM. The numbers of individual sets of MT evaluated (in duplicate) for each factor are given in the order ND-II/ND-MI/T2D-II/T2D-MI): GRO (11/12/10/12), IL6 (12/12/12/13), IL8 (11/12/12/15), IL15 (5/5/5/5/), MCP1 (6/6/7/7), VEGF (8/8/4/4/), BDNF (4/4/5/5), TGFβ1 (3/3/5/5). *p < 0.05 vs. matched control, **p < 0.01 vs. matched control, †*p* < 0.05 vs. matched response to II in MT from the same subject.

In contrast to the relatively modest effects of the II condition on markers of inflammation and insulin action in ND-MT, the same treatment over 24 h induced large increases in the secretion of selected myokines (Figure 6). Meanwhile, MI conditions did not significantly alter secretion of any of the same factors but for TGFβ1. T2D-MTs also responded to II treatment with stimulation of myokine secretion, significantly so for GRO, but with tendencies for IL6 (*p* = 0.0696) and IL8 (*p* = 0.0681). The MI condition was without effect on T2D-MTs for secretion of the myokines measured.

### Impact of Infectious and Metabolic Inflammation on INS-1 Cells

Before evaluating the impact of II and MI stress-induced secreted factors on β-cell function, it was necessary to investigate the direct effects of the II and MI conditions, since those elements would still be present in the MT-CM. INS-1 cells were treated for 24 h with RPMI + αMEM containing either LPS (II) or the glucose/insulin/palmitate mix (MI), processed in the same way as MT-CM. Neither the II nor MI media had a significant effect on total cell protein or caspase 3 cleavage, and only a modest increase in LDH release, while function (total insulin content, insulin release in the presence of either low or high [glucose], GSIS, or IS_max_) was unaltered (Supplementary Figure S3). Thus, any effects of MT-CM on INS-1 cells should be ascribed to factors generated by MT and not the other components of the media. Moving forward, the controls for II-MT-CM or MI-MT-CM, were the 3:1 mix containing either αMEM + II not conditioned by MT or αMEM + MI not conditioned by MT, respectively.

### Effects of Stress-Induced Secreted Factors on INS-1 Cells

#### Insulin Secretion

**FIGURE 7:**
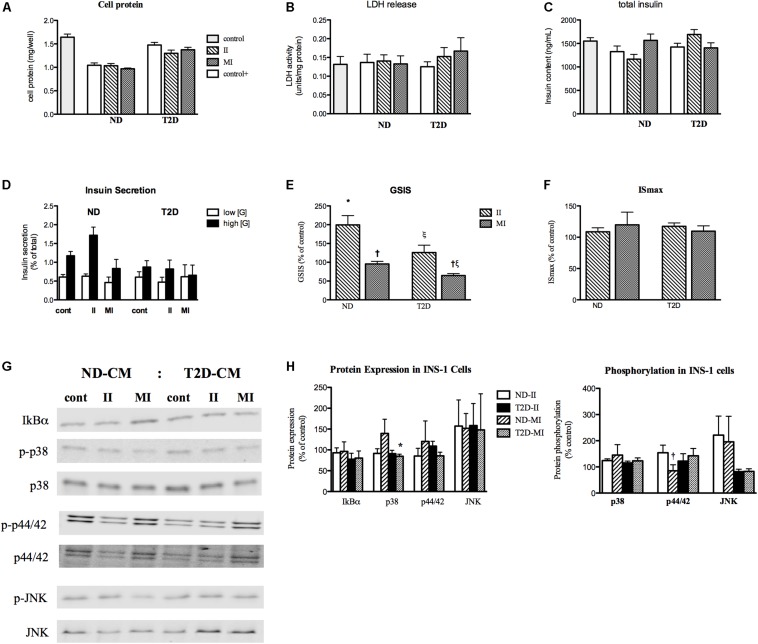
Effects of CM from ND- and T2D-MT challenged with II and MI conditions on INS-1 cell function. INS-1 cells treated with the indicated CM for 24 h before harvest or assay. **(A)** Total cell protein (*n* = 12–24). **(B)** LDH release (*n* = 12–24). **(C)** Total insulin content (*n* = 12–24). **(D)** Insulin secretion (*n* = 10–14). **(E)** GSIS (*n* = 10–14). **(F)** IS_max_ (*n* = 8–14). **(G)** Representative western blots for IkBα, total and phosphorylated p38, p44/42, and JNK. **(H)** Quantization of western blots (*n* = 4–8). Results presented as absolute value or as a percentage of the appropriate control, II or MI non-conditioned media. Ave + SEM. Panels **(A–C)**; Control = RPMI: a-MEM (3:1) w/o treatment conditioned by MT from the same individual, control+ = RPMI: a-MEM (3:1) + II or MI not conditioned by MT. Panels **(D,E,G)**, control = RPMI: a-MEM (3:1) w/o treatment conditioned by MT from the same individual. *p < 0.05 vs. control, †*p* < 0.05 vs. II.

CM derived from either ND- or T2D-MT treated under the II condition had no effect on any of the markers of INS-1 cell viability (Figures 7A,B and Supplementary Figure S3C). Total insulin content was also unaltered (Figure 7C). However, after treatment with II-ND-CM, GSIS was doubled (100 ± 25% of control), compared to the relevant non-conditioned media control (Figure 7D): CM from T2D MT (II-T2D CM) showed no such effect. This effect of II-ND-CM was limited to GSIS, as both IS in the presence of low [glucose] (Figure 7) and IS_max_ (Figure 7E) were unaltered.

CM from MI-treated ND- and T2D-MT also had no effect on INS-1 cell viability or total insulin content (Figures 7A,B and Supplementary Figure S3C). In contrast to the response to the II condition, MI-ND-CM had no effect on either GSIS or IS_max_ (Figures 7E,F). However, exposure to the CM from T2D-MT (MI-T2D-CM) reduced GSIS, to 65 ± 5% of control (*p* < 0.05) (Figure 7D). Again, IS in the presence of low [glucose] (Figure 7D) and IS_max_ (Figure 7F) were unperturbed.

IkBα content, was not altered by either II or MI/ND-CM or II or MI/T2D-CM (Figures 7G,H). Similarly, the various CM had only modest, or no, effects on inflammatory signaling (Figure 7H): only an effect of MI/T2D-CM to reduce p38 protein (by 10%) attained statistical significance.

### Infectious and Metabolic Inflammation Regulation of Insulin Secretion

In studying further the nature of regulation of β-cell function by MT-secreted factors, we focused on the two conditions where GSIS was altered; treatment with II-ND-CM and MI-T2D-CM.

**FIGURE 8:**
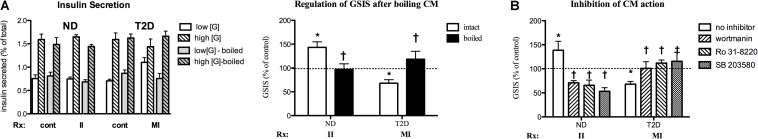
Characterization of MT-CM regulation of GSIS. **(A)** Cells treated for 24 with intact MT-CM or MT-CM boiled before exposure: Left panel – insulin secretion, Right panel – GSIS (*n* = 10). **(B)** Inhibition. Cells treated with the indicated CM in the absence or presence of SB203580 (100 nM, *n* = 6 for ND/5 for T2D), Ro 31-8220 (50 nM, *n* = 6/5), or wortmannin (100 nM, *n* = 6/8) before GSIS determined. Control = RPMI: αMEM (3:1) w/o treatment conditioned by MT from the same individual. *p < 0.05 vs. matched control, †*p* < 0.05 vs. intact media **(A)** or no inhibitor **(B)**.

As one step in determining the potential nature of the active factor(s) responsible for regulation of GSIS, INS-1 cells were cultured for 24 h with the indicated MT-CM that had either been left unprocessed or boiled for 10 min before treating cells. Boiling fully abrogated stimulation of GSIS by II-ND-CM or the suppression by MI-T2D-CM (Figure 8A), suggesting that the factor(s) responsible for the effects on GSIS are protein in nature and therefore, by definition, can be termed myokines.

The signaling pathways involved in myokine regulation of β-cell function were investigated by simultaneous treatment of INS-1 cells with specific MT-CM and inhibitors of PI3K (wortmannin), PKC (Ro 31-8220) and p38 MAPK (SB 203590) for 24 h. All three of the inhibitors were able to fully reverse II-ND-CM stimulation of GSIS (Figure 8B). Indeed, each inhibitor was also able to suppress GSIS below control. Conversely, each of the inhibitors restored GSIS after MI-T2D-CM treatment (Figure 8B). These results suggest that multiple pathways, and potentially multiple factors, participate in MT regulation of GSIS.

## Discussion

The defining characteristics of T2D are insulin resistance in peripheral tissues and, ultimately, β-cell failure (DeFronzo and Tripathy, 2009). The importance of insulin resistance in SkM itself is well known (reviewed in DeFronzo and Tripathy, 2009). More recently, attention has been paid to a role for SkM in regulating β-cell mass and function (Shirakawa et al., 2017). Such regulation could be mediated by myokines, protein factors secreted from SkM (Barlow and Solomon, 2018). For example, exercise is known to improve/protect β-cell viability (Narendran et al., 2017; Paula et al., 2018) at least in part through IL6 released from SkM, either acting directly on β-cells (Christensen et al., 2015; Paula et al., 2015), or by enhancing GLP-1 secretion from L cells and alpha cells (Ellingsgaard et al., 2011). Yet, others have found no effects of physiologic levels of IL6 on insulin secretion (Barlow et al., 2018). Other exercise-induced factors that have been suggested to improve β-cell mass and/or function include irisin (Natalicchio et al., 2017), RANTES (Mizgier et al., 2017) and Fractalkine (CX3CL1) (Barlow and Solomon, 2018).

The muscle secretome could also exert negative effects on β-cell mass and function. For example, CM from myotubes from healthy subjects where an insulin resistant state was induced by treatment with TNFα proved capable of reducing both β-cell mass and GSIS (Bouzakri et al., 2011), while IL13 protected islets against cytokine-induced apoptosis, with no effects on GSIS (Rutti et al., 2015). Conversely, while feeding of a high fat diet (20% palmitate) made male C57BL/6 mice insulin resistant, exosome-like vesicles isolated from muscle of these animals were able to induce proliferation of MIN6B1 cells and isolated islets (Jalabert et al., 2016), possibly to compensate for the insulin resistant state. These authors implicated miR-16 in this action (Jalabert et al., 2016).

To date, investigations of SkM effects on β-cell viability and function have focused on tissue or cells from healthy animals or humans, manipulated by exercise, diet, or other treatments to induce insulin resistance, but not on the impact of T2D itself. Since we, and others have reported that altered secretion at the protein (Brandt et al., 2012; Ciaraldi et al., 2016), miRNA (Massart et al., 2016, 2017), and metabolite levels (Rai and Demontis, 2016), is an intrinsic property of muscle in T2D (reviewed in Eckardt et al., 2014; Garneau and Aguer, 2019), we sought to determine if β-cell/muscle communication was altered in T2D. To that end, we employed the human SkM cell system (Henry et al., 1995). Multiple investigators have shown that human SkM satellite cells, when cultured and differentiated to myotubes display many of the properties of intact muscle, including T2D-related differences in glucose and fat metabolism (reviewed in Aas et al., 2013; Gaster, 2018), protein expression (Al-Khalili et al., 2014) and, most important to the current goals, secretory profile (Ciaraldi et al., 2016; Massart et al., 2016). Media conditioned by culture with myotubes, was taken as a surrogate for secretion into the extracellular space, and ultimately into the circulation.

When cultured under standard conditions, with glucose and insulin levels similar to those present in the circulation of ND subjects, CM from neither ND nor T2D-MT had any effects, over 24 h, on INS-1 cell viability and function (Supplementary Figure S1), or on markers of inflammation. Thus, the T2D-related intrinsic differences in secretion of myokines and other factors we, and others have reported (Ciaraldi et al., 2016; Massart et al., 2016), alone would not appear to contribute to changes in β-cell mass or regulated insulin secretion over this time frame. Unlike our finding of no effect of ND-MT-CM on GSIS in INS-1 cells, Bouzakri et al. (2011) reported that media conditioned by MT from healthy subjects increased GSIS in primary rat β-cells. Multiple differences in experimental conditions, including the target cell itself might account for this difference. It is critical to note that the control environment in which the MT were cultured and CM generated represents, at least for T2D-MT, an artificial situation. For that reason, we elected to challenge myotubes under conditions that more closely modeled the environment seen *in vivo* in T2D individuals. To mimic the chronic, low-grade systemic inflammation present in T2D (Olefsky and Glass, 2010), we chose treatment with LPS (II), a widely employed intervention (reviewed in Boutagy et al., 2016). While LPS treatment stimulated secretion of a number of pro-inflammatory myokines (Figure 6) from both ND and T2D-MT, this was accompanied by only modest, if any, changes in cellular indicators of inflammation or inflammatory signaling in the myotubes. Insulin signaling through Akt phosphorylation was also unaltered by the II condition.

Meanwhile, as might be expected, the hyperglycemic/hyperinsulinemic/hyperlipidemic T2D-like conditions induced insulin resistance at the level of Akt phosphorylation in ND-MT, but did not exacerbate the already impaired Akt response in T2D-MT; it may be that even more severe (non-physiologic) conditions would be needed to induce even more severe insulin resistance for this response. ND- and T2D-MT differed in a number of other ways in their responses to the MI environment. Stimulation of myokine secretion, while modest compared to the II response, was seen for a number of factors from T2D-MT, unlike with ND-MT, where only TGFβ1 secretion was altered significantly. These results reveal another aspect of the T2D phenotype in muscle that is retained in MT, an increased sensitivity to a hyperglycemic/hyperinsulinemic/hyperlipidemic environment.

Given the different conditions under which human myotubes and INS-1 cells, the established β-cell model employed for these studies, are cultured, considerable effort had to be put into determining how the MT media might influence INS-1 cells, even in the absence of contributions from MTs. Fortunately, only minor modifications in serum-free α-MEM were needed to maintain INS-1 cell viability and function (Supplementary Figure S1). Furthermore, the II and MI conditions used to treat MT to generate CM had only modest direct effects on the outcomes of interest in INS-1 cells, which were accounted for by inclusion of the appropriate controls. Thus, any effects of II- or MI-MT-CM on INS-1 cell mass and/or function could be ascribed to contributions from the ND and T2D myotubes.

The major findings of the current studies are those presented in Figures 6, 7; that MT from ND and T2D subjects respond differently to the challenges represented by infectious and metabolic inflammation with regard to both their secretome, and the effects of those secretions on β-cell function. Furthermore, these differential effects are highly specific for glucose regulated insulin secretion, as neither markers of β-cell mass, insulin production, nor maximal insulin secretion, were impacted. While ND-MT are able to produce and secrete factor(s) that augment (II) or possibly protect (MI) GSIS, T2D-MT lacked these capacities.

Further studies provided additional information about the nature of the mechanisms by which factors secreted from ND- and T2D-MT influence GSIS. We learned that both the beneficial (II-ND-CM) and negative (MI-T2D-CM) effects are mediated by protein factors (Figure 8A) and therefore could be considered to be myokines. Another common feature of the two responses is that multiple signaling pathways; PI3-K, PKC and p38 MAPK, are involved (Figure 8B).

In trying to identify the specific factors present in CM responsible for regulation of GSIS one would look for those that are uniquely altered in II-ND-CM and MI-T2D-CM. Such factors would need to meet the following criteria: (1) differentially regulated in ND-CM under II vs. control or MI conditions, (2) differentially regulated in T2D-CM under MI vs. control or II condition, (3) changed in ND- but not T2D-CM under the II condition, (4) changed in T2D- but not ND-CM under MI condition. Of the proteins measured (Figure 6), no individual protein met all of the necessary criteria. The implication that multiple signaling pathways mediate these specific instances of modulation of regulated insulin secretion (Figure 8B) suggests that multiple factors might be involved, which was not considered in our initial analysis of the data.

A number of muscle-derived factors have been shown to modulate GSIS: irisin (Natalicchio et al., 2017), IL6 (Ellingsgaard et al., 2011; Christensen et al., 2015), fractalkine (Barlow and Solomon, 2018), angiogenin, and osteoprotegerin (Rutti et al., 2018) and Wnt3a (Kozinski et al., 2016); RANTES improved basal insulin secretion, but was without effect on GSIS (Mizgier et al., 2017). The involvement of any of these factors in the responses described in the current report might be questioned, as many of these same factors also protected the β-cell or islet systems studied from apoptosis, while we found no such effects of specific MT-CM on caspase 3 cleavage, an accepted marker of apoptosis. However, comparisons between studies are complicated by the use of different challenges for muscle cells; palmitate alone (Natalicchio et al., 2017), insulin alone (Mizgier et al., 2017), or selected cytokines (Christensen et al., 2015; Rutti et al., 2015, 2018). Also, none of these studies evaluated MT from individuals with T2D. Obviously additional studies, possibly proteomic analysis of specific CM, are needed to identify the factor(s) responsible for the regulation of GSIS reported here.

While Kozinski et al. (2016) reported that reciprocal changes in Wnt3a and Wnt4 secretion from adipose tissue and SkM tissue and C2C12 cells could influence islet proliferation and GSIS, we were unable to detect either protein in MT-CM, even after concentration. Several factors could contribute to such a discrepancy in the results. In intact SkM, Wnts are found in axons and satellite cells, where they play a role in differentiation (Cisternas et al., 2014). It is possible that Wnt expression could be lost with terminal differentiation of human myotubes, while C2C12 cells may retain aspects of the myoblast phenotype. However, that does not rule out a role for the Wnt system in mediating β-cell adaptations to specific stressors, merely that, in humans, adipose tissue and not muscle may represent the primary source of Wnt signaling proteins.

The relationship between chronic low-grade inflammation in adipose tissue and insulin resistance in obesity and T2D is well established (reviewed in DeFronzo and Abdul-Ghani, 2011); the situation is less clear with regard to SkM, as there are reports of both normal (Tam et al., 2012; Amazou et al., 2016; Perry et al., 2016) and elevated (Fink et al., 2013; Patsouris et al., 2014; Brown et al., 2015) markers of inflammation in SkM from obese insulin-resistant and T2D individuals. Under the normoglycemic/insulinemic/low lipid, control culture conditions employed in this study we found no differences between ND and T2D MT for multiple markers of inflammation, even as T2D-MT displayed a tendency (this report) to secrete elevated levels of several pro-inflammatory cyto- and chemokines (Figure 2). These results suggest that inflammation in SkM may not be an effect autonomous to myotubes, but due to the recruitment of pro-inflammatory cells into SkM.

Conversely, it has been reported that MT from T2D subjects display elevated phosphorylation of p38 MAPK (Brown et al., 2015). Yet, in that instance the indication of inflammation was uncoupled from insulin resistance, as glucose uptake was unaltered (Brown et al., 2015). A similar uncoupling of SkM inflammation and insulin resistance has been seen in obese subjects (Amazou et al., 2016), after SkM-specific over-expression of MCP-1 (Evers-van Gogh et al., 2016), and with palmitate treatment of L6 myotubes (Sinha et al., 2004). While we found that LPS treatment of MT resulted in increased secretion of multiple pro-inflammatory factors, the finding of only modest changes in markers of inflammation is additional support for the importance of non-MT cells to the establishment of inflammation in SkM. Most interesting are the responses of MT to the diabetic-like, MI conditions, where in ND-MT there was minimal or no impact on either secretion of the myokines measured or markers of inflammation, which would be consistent with the lack of effects of MI-ND-CM on insulin secretion, while the ability of MI-T2D-CM to suppress GSIS was accompanied by modest changes in p38 and p44/42 MAPK protein expression and phosphorylation. The differing responses to the II and MI treatments reveal additional aspects of the T2D phenotype that are intrinsic to SkM, as they are retained in culture.

Several limitations in the current studies require mention. One is the nature of the human SkM cell system. While, as mentioned earlier, these cells reflect many of the properties of their donors, they also represent denervated muscle. Denervation in itself can induce insulin resistance (Turinsky and DAmrau-Abney, 1998), while removing the contributions of central, neural, control of metabolism, which could also be a site of difference between healthy individuals and those with T2D (Porte et al., 2005). Furthermore, while inflammatory cytokines have been shown to inhibit myoblast differentiation, this effect was not present on myotubes (Langen et al., 2001); we avoided this effect by treating fully differentiated myotubes. An advantage of the human MT system is that it permits study of the cell-autonomous effects of MT on β-cell mass/function, yet it neglects the contributions of other cell types, such as pro- and anti-inflammatory cells infiltrating SkM (see above). Another difference from whole body physiology is the static nature of the current studies, both for generation of CM and treatment of INS-1 cells. In addition, we utilized confluent INS-1 cells, which while that would allow evaluation of myokine effects on cell viability, including apoptosis, it precludes following effects on proliferation, limiting comparisons to some of the other reports on myokine effects on β-cells.

In summary, we report here, in agreement with the work of others, that factors secreted from SkM can influence regulated insulin secretion. Novel information includes the observation that MT from individuals with T2D differ from MT from ND subjects with regard to the factors they secrete in response to conditions modeling aspects of the environment present in T2D. While ND-MT are able to protect or augment GSIS when faced with these challenges, T2D-MT lack these compensatory responses. Rather, under T2D-like conditions (MI), T2D-MT act in a manner to impair GSIS, which, together with the impaired metabolism and insulin resistance intrinsic to T2D SkM, would contribute in multiple ways to the metabolic dysfunction characteristic of T2D. Under the conditions investigated in this report, muscle secretome regulation of GSIS is mediated by currently unknown protein factors that act through both pro-inflammatory (p38 MAPK) and other (PI3-K and PKC) signaling pathways.

## Data Availability Statement

The datasets generated for this study are available on request to the corresponding author.

## Ethics Statement

The experimental protocol was approved by the Human Research Protection Programs of the Veterans Affairs San Diego Healthcare System and the University of California, San Diego. Informed written consent was obtained from all subjects after explanation of the protocol.

## Author Contributions

AR and TC conceived and designed the study, performed the experiments, researched the data, and wrote the manuscript. RH contributed to study design, reviewed and edited the manuscript. TC is the guarantor of this work and, as such, had full access to all of the data in the study and takes full responsibility for the accuracy of the data analysis.

## Conflict of Interest

The authors declare that the research was conducted in the absence of any commercial or financial relationships that could be construed as a potential conflict of interest.

## Supplementary Material

FIGURE S1 Effects of culture media on INS-1 cell viability and function. INS-1 cells treated with the indicated media, RPMI1640 or RPMI+ the indicated proportion of αMEM, for 24 h before harvest or assay. **(A)** Total cell protein (*n* = 12). **(B)** LDH release (*n* = 9). **(C)** Representative western blot for total and cleaved caspase 3: 1 – RPMI1640, 2 – αMEM, 3–5 – T2D-MT-CM, 6–8 – ND-MT-CM. **(D)** Total, secreted and cell-associated, insulin content (*n* = 12). **(E)** GSIS (*n* = 10). **(F)** IS_max_ (*n* = 5). For panels **(D–F)**, αMEM = 3:1 RPMI + αMEM. **p* < 0.05 vs. paired control.

FIGURE S2 Effects of II and MI conditions on MT viability. MT treated for 48 h (cell harvesting). **(A)** Total cell protein (*n* = 12–14). **(B)** Representative western blot for total and cleaved caspase 3: cont – Jurkat cell extract treated + cytochrome C, αMEM = 3:1 RPMI + αMEM not conditioned, +II = 3:1 RPMI + (αMEM not conditioned + LPS), + MI = 3:1 RPMI + (αMEM not conditioned + MI mix).

FIGURE S3 Effects of II and MI media on INS-1 cell viability and function. INS-1 cells treated with the indicated media for 24 h before harvest or assay. **(A)** Total cell protein (*n* = 10–12). **(B)** LDH release (*n* = 10–12). **(C)** Representative western blots for total and cleaved caspase 3: I – non-conditioned αMEM, 1 – control, 2 – +II, 3 – +MI; II – ND-MT-CM, III – T2D-MT-CM; cont – Jurkat cell extract treated + cytochrome C. **(C)** Total, secreted and cell-associated, insulin content (*n* = 10–11). **(D)** Insulin secretion (*n* = 7–10). **(E)** GSIS (*n* = 7–10). **(F)** IS_max_ (*n* = 8–12). **p* < 0.05 vs. paired control.
