# Supplementary figures and images for: Myokine Regulation of Insulin Secretion: Impact of Inflammation and Type 2 Diabetes
Source: Front Physiol. 2020 Jan 22;10:1608. doi: 10.3389/fphys.2019.01608 (PMC6987462; doi:10.3389/fphys.2019.01608)

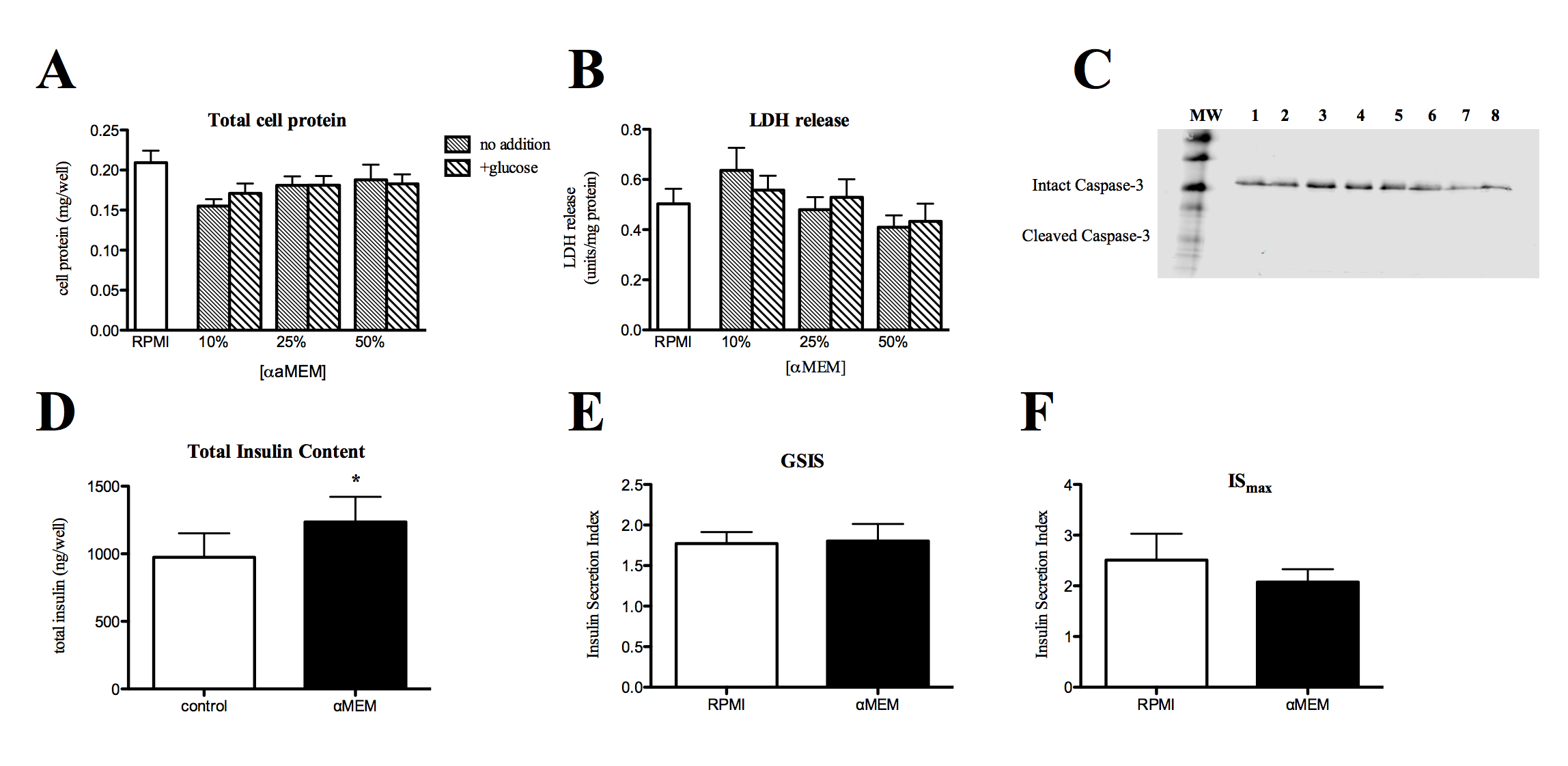

Supplement: FIGURE S1 — Effects of culture media on INS-1 cell viability and function. INS-1 cells treated with the indicated media, RPMI1640 or RPMI+ the indicated proportion of αMEM, for 24 h before harvest or assay. (A) Total cell protein (n = 12). (B) LDH release (n = 9). (C) Representative western blot for total and cleaved caspase 3: 1 – RPMI1640, 2 – αMEM, 3–5 – T2D-MT-CM, 6–8 – ND-MT-CM. (D) Total, secreted and cell-associated, insulin content (n = 12). (E) GSIS (n = 10). (F) ISmax (n = 5). For panels (D–F), αMEM = 3:1 RPMI + αMEM. *p < 0.05 vs. paired control. [file Image_1.tiff]

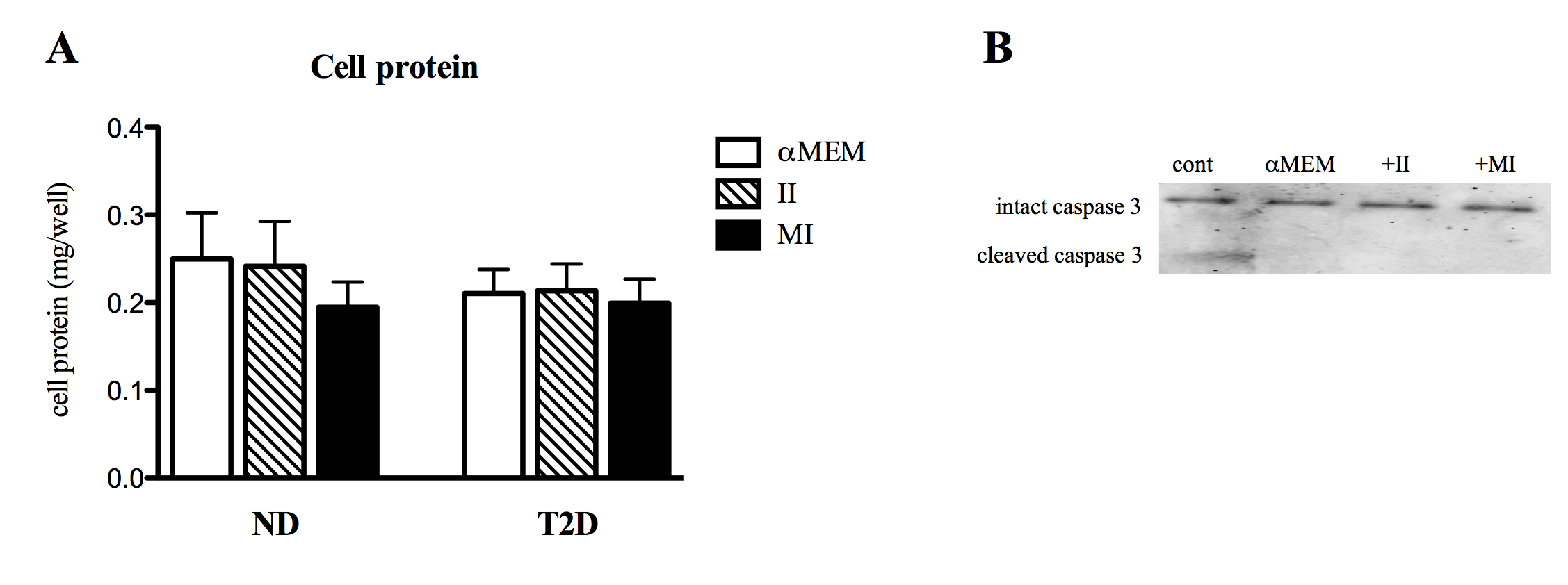

Supplement: FIGURE S2 — Effects of II and MI conditions on MT viability. MT treated for 48 h (cell harvesting). (A) Total cell protein (n = 12–14). (B) Representative western blot for total and cleaved caspase 3: cont – Jurkat cell extract treated + cytochrome C, αMEM = 3:1 RPMI + αMEM not conditioned, +II = 3:1 RPMI + (αMEM not conditioned + LPS), + MI = 3:1 RPMI + (αMEM not conditioned + MI mix). [file Image_2.tiff]

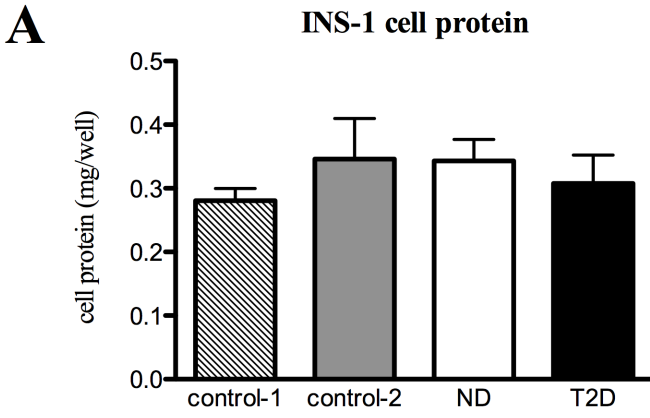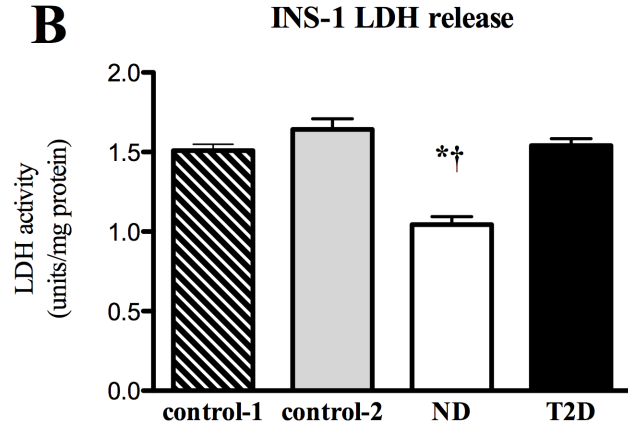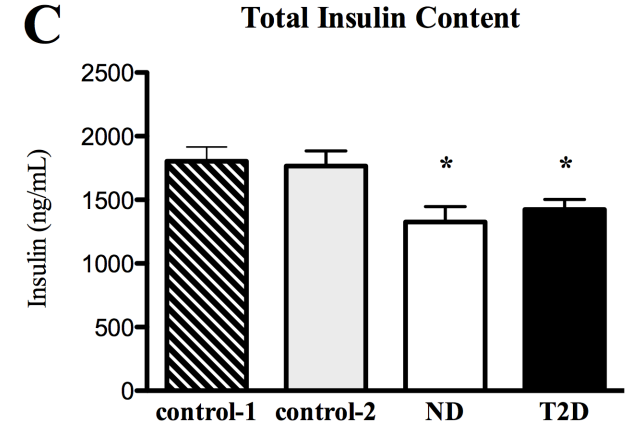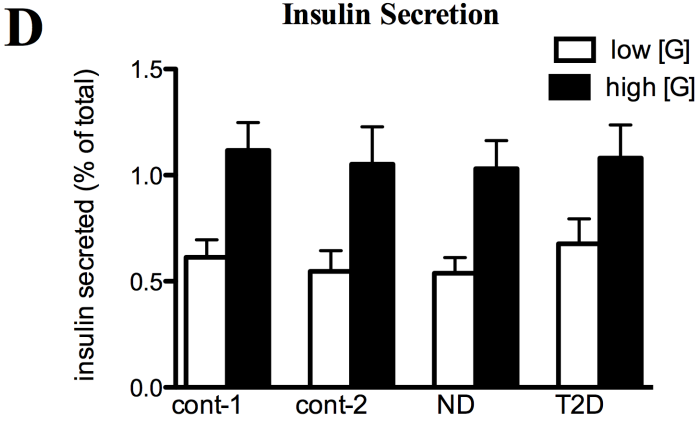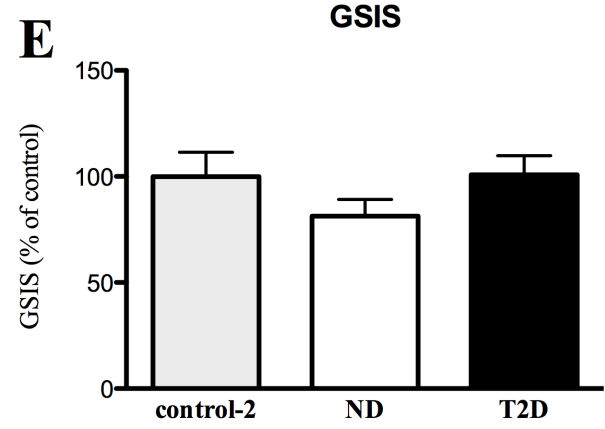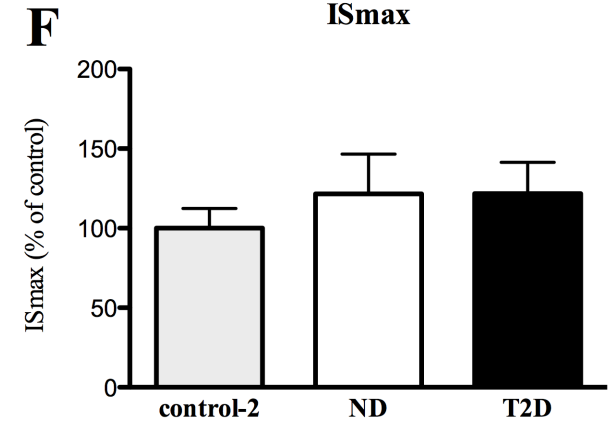

Supplement: FIGURE S3 — Effects of II and MI media on INS-1 cell viability and function. INS-1 cells treated with the indicated media for 24 h before harvest or assay. (A) Total cell protein (n = 10–12). (B) LDH release (n = 10–12). (C) Representative western blots for total and cleaved caspase 3: I – non-conditioned αMEM, 1 – control, 2 – +II, 3 – +MI; II – ND-MT-CM, III – T2D-MT-CM; cont – Jurkat cell extract treated + cytochrome C. (C) Total, secreted and cell-associated, insulin content (n = 10–11). (D) Insulin secretion (n = 7–10). (E) GSIS (n = 7–10). (F) ISmax (n = 8–12). *p < 0.05 vs. paired control. [file Image_3.pdf]
